# Reduced dietary salt for the prevention of cardiovascular disease

**DOI:** 10.1590/1516-3180.20151333T2

**Published:** 2014-12-19

**Authors:** Alma J. Adler, Fiona Taylor, Nicole Martin, Sheldon Gottlieb, Rod S. Taylor, Shah Ebrahim

## Abstract

**BACKGROUND:**

This is an update of a Cochrane review that was first published in 2011 of the effects of reducing dietary salt intake, through advice to reduce salt intake or low-sodium salt substitution, on mortality and cardiovascular events.

**OBJECTIVES:**

1. To assess the long-term effects of advice and salt substitution, aimed at reducing dietary salt, on mortality and cardiovascular morbidity. 2. To investigate whether a reduction in blood pressure is an explanatory factor in the effect of such dietary interventions on mortality and cardiovascular outcomes.

**METHODS:**

*Search methods:* We updated the searches of CENTRAL (2013, Issue 4), MEDLINE (OVID, 1946 to April week 3 2013), EMBASE (OVID, 1947 to 30 April 2013) and CINAHL (EBSCO, inception to 1 April 2013) and last ran these on 1 May 2013. We also checked the references of included studies and reviews. We applied no language restrictions.

*Selection criteria:* Trials fulfilled the following criteria: (1) randomised, with follow-up of at least six months, (2) the intervention was reduced dietary salt (through advice to reduce salt intake or low-sodium salt substitution), (3) participants were adults and (4) mortality or cardiovascular morbidity data were available. Two review authors independently assessed whether studies met these criteria.

*Data collection and analysis:* A single author extracted data and assessed study validity, and a second author checked this. We contacted trial authors where possible to obtain missing information. We extracted events and calculated risk ratios (RRs) and 95% confidence intervals (CIs).

**MAIN RESULTS:**

Eight studies met the inclusion criteria: three in normotensives (n = 3518) and five in hypertensives or mixed populations of normo- and hypertensives (n = 3766). End of trial follow-up ranged from six to 36 months and the longest observational follow-up (after trial end) was 12.7 years.

The risk ratios (RR) for all-cause mortality in normotensives were imprecise and showed no evidence of reduction (end of trial RR 0.67, 95% confidence interval (CI) 0.40 to 1.12, 60 deaths; longest follow-up RR 0.90, 95% CI 0.58 to 1.40, 79 deaths n = 3518) or in hypertensives (end of trial RR 1.00, 95% CI 0.86 to 1.15, 565 deaths; longest follow-up RR 0.99, 95% CI 0.87 to 1.14, 674 deaths n = 3085).

There was weak evidence of benefit for cardiovascular mortality (hypertensives: end of trial RR 0.67, 95% CI 0.45 to 1.01, 106 events n = 2656) and for cardiovascular events (hypertensives: end of trial RR 0.76, 95% CI 0.57 to 1.01, 194 events, four studies, n = 3397; normotensives: at longest follow-up RR 0.71, 95% CI 0.42 to 1.20, 200 events; hypertensives: RR 0.77, 95% CI 0.57 to 1.02, 192 events; pooled analysis of six trials RR 0.77, 95% CI 0.63 to 0.95, n = 5912). These findings were driven by one trial among retirement home residents that reduced salt intake in the kitchens of the homes, thereby not requiring individual behaviour change.

Advice to reduce salt showed small reductions in systolic blood pressure (mean difference (MD) -1.15 mmHg, 95% CI -2.32 to 0.02 n = 2079) and diastolic blood pressure (MD -0.80 mmHg, 95% CI -1.37 to -0.23 n = 2079) in normotensives and greater reductions in systolic blood pressure in hypertensives (MD -4.14 mmHg, 95% CI -5.84 to -2.43 n = 675), but no difference in diastolic blood pressure (MD -3.74 mmHg, 95% CI -8.41 to 0.93 n = 675).

Overall many of the trials failed to report sufficient detail to assess their potential risk of bias. Health-related quality of life was assessed in one trial in normotensives, which reported significant improvements in well-being but no data were presented.

**AUTHORS’ CONCLUSIONS:**

Despite collating more event data than previous systematic reviews of randomised controlled trials, there is insufficient power to confirm clinically important effects of dietary advice and salt substitution on cardiovascular mortality in normotensive or hypertensive populations. Our estimates of the clinical benefits from advice to reduce dietary salt are imprecise, but are larger than would be predicted from the small blood pressure reductions achieved. Further well-powered studies would be needed to obtain more precise estimates. Our findings do not support individual dietary advice as a means of restricting salt intake. It is possible that alternative strategies that do not require individual behaviour change may be effective and merit further trials.

**The abstract of this review is available from:** http://onlinelibrary.wiley.com/doi/10.1002/14651858.CD009217.pub3/abstract
